# At the dawn of delegation? Experiences and attitudes of general practitioners in Germany – a questionnaire survey

**DOI:** 10.1186/s12875-017-0697-y

**Published:** 2017-12-19

**Authors:** Katja Goetz, Anna Kornitzky, Janis Mahnkopf, Jost Steinhäuser

**Affiliations:** Institute of Family Medicine, University Hospital Schleswig-Holstein, Campus Luebeck, Ratzeburger Allee 160, 23538 Luebeck, Germany

**Keywords:** General practitioners, Job satisfaction, Legal aspect, Non-clinical staff, Qualification, Task shifting

## Abstract

**Background:**

In the future, ‘delegation’ as task shifting from general practitioners (GPs) to non-physicians will be important in primary care. Therefore, the aim of this study was to evaluate the attitudes towards the concept of task shifting and to identify predictors of a positive attitude towards task shifting from the perspective of GPs.

**Methods:**

This cross-sectional questionnaire study analysed attitudes towards the concept of task shifting and delegated tasks from the perspective of GPs who were recruited in the German federal state of Schleswig-Holstein. Descriptive statistics and binary regression analyses were computed to identify potential predictors of a positive attitude towards task shifting.

**Results:**

Out of 1538 questionnaires distributed, 577 GP questionnaires were returned (response rate: 37.5%). A total of 53.2% of the respondents were male, and 37.3% were female. A positive attitude regarding task shifting was shown by 49% of the participating GPs. The highest level of agreement (95.2%) was found for time savings with task shifting, and a lower agreement (39%) was found regarding the lack of clarity concerning the responsibilities and legal aspects with regards to task shifting. The most frequently delegated tasks were recording electrocardiograms and measuring blood glucose levels. A positive attitude towards task shifting was positively associated with higher job satisfaction and a need for qualified staff.

**Conclusion:**

Our sample of GPs for this study was very open-minded towards the concept of task shifting. Germany is just beginning this delegation, but the implementation of task shifting depends on different aspects, such as legal requirements, adequate payment and qualified staff. Finally, there is a need for continuing professional development in primary care teams, especially for non-clinical practice staff.

**Electronic supplementary material:**

The online version of this article (10.1186/s12875-017-0697-y) contains supplementary material, which is available to authorized users.

## Background

The concept of task shifting is an important development within primary care systems in industrialized countries. International experiences have shown that, besides physicians, other non-physician health professionals, e.g., nurse practitioners or registered nurses, support the medical care of patients [1, 2]. However, the degree of task shifting differs substantially among countries. A systematic review has revealed that the evidence from good quality studies concerning task shifting from physicians to nurses is very limited [3]. Different political and legal frameworks in the respective countries are responsible for task shifting from physicians to non-physicians. In comparison with countries such as Canada, New Zealand and the Netherlands, Germany appears to be less advanced in adopting task shifting [1].

The shifting of medical tasks to non-physician healthcare staff has been allowed in Germany since 2008. One of these medical tasks is the delegation of home visits to non-physician staff, which is completely legal [4]. Therefore, in Germany, different delegation projects have been initiated, such as VERAH (health care assistants in family practices) and AGnES (general practitioner (GP)-supporting, community-based, e-health-assisted, systematic intervention) [5, 6]. The AGnES project has been well evaluated and has been implemented in two federal states (Mecklenburg-Vorpommern and Brandenburg). This concept is based on GP support in the context of home visits [7]. An economic evaluation of the AGnES-practice assistants has revealed a decrease in home visits performed by GPs [5]. Hence, it can be assumed that GPs have more time for other activities within their practices. Moreover, most GPs participating in previous studies have agreed that task shifting saves the physicians’ time and relieves them of some of their workload, especially that concerning home visits [6, 8].

Therefore, task shifting as a transfer of clinical tasks from a physician to another health care staff member is crucial for health care teams consisting of physicians and non-physicians [2]. Moreover, high-performing team-based care could be associated with the redistribution of tasks in addition to the shifting of tasks from clinicians to non-clinicians [9].

In Germany, a country where task shifting is in an emerging phase, the perceptions of the GPs regarding the benefits and concerns of task shifting have been unknown. The German Medical Association has provided a list of different tasks that can be delegated from physician to non-physician staff [10], but the experiences and attitudes from the physicians’ perspectives and the aspects that might support a positive attitude towards task shifting have not been well evaluated. Therefore, the aim of the current study was to evaluate the experiences and attitudes towards the concept of task shifting and to identify predictors of a positive attitude towards task shifting from the perspective of GPs.

## Methods

### Design and participants

The study conformed to the STROBE-Guidelines (Strengthening the Reporting of Observational Studies in Epidemiology) [11]. This cross-sectional study was conducted in Schleswig-Holstein, a federal state in northern Germany. Schleswig-Holstein is a state with a low population density that is located between the North and Baltic Seas and can be characterized as a rural area. The population density was over 181 inhabitants per square kilometre in 2016. The rate of unemployment was 5.8% as of June 2017 [12].

A total of 1538 questionnaires were sent to all GPs of the federal state of Schleswig-Holstein by mail. In Germany, the term ‘GP’ includes general internists and general practitioners. Therefore, there was no differentiation between these groups in the questionnaire. As an alternative for completing the questionnaire, there was a short “non-response-sheet” that could be completed and returned instead. The addresses of the GPs’ practices were obtained from the website ‘https://arztsuche.kvsh.de/’ of the regional Association of Statutory Health Insurance Physicians. No reminders were sent out. The survey was conducted between June and July 2015. Because this was an explorative study, no power calculation was determined. The return of the anonymous paper-based questionnaire was classified as informed consent. The questionnaire of this survey was added as supplementary file.

### Measurement

Personal and practice characteristics were measured in the questionnaire, including gender, age, duration of employment in the practice and the location of the practice (on the basis of the car registration number of the administrative district). Duration of employment in the practice was grouped into four categories: ‘less than 5 years’, ‘5 to 10 years’, ‘11 to 20 years’ or ‘more than 20 years’. The location of the practice was measured on the basis of the car registration number and was grouped into urban area, medium-sized town and rural area on the basis of the BBSR (Federal Institute for Research on Building, Urban Affairs and Spatial Development) [13]. Furthermore, measurements of the number of GPs per practice, the number of physician assistants per practice and the number of home visits per week were also taken into account. The benefits and concerns towards the concept of task shifting in general were evaluated with a self-developed questionnaire based on a literature review. The attitudes towards the concept of delegation were evaluated with three response categories: positive attitude, partly positive attitude and negative attitude. For the purpose of analyses, these categories were summarized into two groups: positive attitude and negative attitude (consisting of partly negative and negative attitudes). An additional part of the questionnaire addressed the GPs’ experiences with different delegated tasks. This part was based on a systematic literature review concerning the current state of the concept of task shifting in the German health system and within the international context. The questionnaire evaluated the tasks that have been regulated within the advanced training for non-clinical staff offered by the German Medical Association [10] and could delegated from the physician to non-clinical staff. The clarity of the questionnaire was checked with two GPs, who used the think aloud technique, but the questionnaire was not validated [14].

### Data analysis

Analyses were performed using SPSS 24.0 (SPSS Inc., IBM). Continuous data were summarized using means and standard deviations. Categorical data were presented as frequency counts and percentages. Moreover, the agreements of different benefits and concerns regarding the implementation of the concept of task shifting and the currently delegated tasks were presented as frequency counts and 95% confidence intervals. Furthermore, a binary logistic regression analysis was performed with the binary variable ‘attitude towards the concept of task shifting’ (1 = positive and 0 = negative) as the outcome variable and individual characteristics, benefits and concerns regarding the implementation of the concept of task shifting and the delegated tasks as explanatory variables. The Hosmer–Lemeshow test was used to evaluate the suitability of these logistic regression models [15]. The incidence of missing data <10% was negligible for the data analysis. An alpha level of *P* < 0.05 was used for tests of statistical significance.

### Ethical approval

Ethical approval for this research study was obtained from the University of Luebeck in May 2015 (Approval No. 15–110). No additional data were evaluated.

## Results

### Characteristics of participants

Out of 1538 questionnaires distributed, 577 questionnaires were returned from GPs (response rate: 37.5%). Among the 577 returned questionnaires, 107 were “non-responders”. The participants who responded to the long version questionnaire (*n* = 470) had a mean age of 55.08 years (SD = 8.2). Of these samples, 53.2% of the respondents were male, and 37.3% were female. The non-responders differed significantly in age and gender. More non-responder GPs were female and were older than the respondents. More than half of the respondents (58.4%) had been working as GPs for over ten years, and 35.2% had been working for over 20 years. On average, 17.12 patients per week were visited at home by these GPs. Moreover, the majority of GPs (58.3%) felt that home visits led to a reduced amount of time for other tasks at the practice. A positive attitude towards task shifting was observed by 49% of GPs. The incidence of missing data was <10%. Background information is presented in Table 1.

**Table 1:** Characteristics of GPs and their practices (*n* = 577)

Characteristics		Responder (*n* = 470)	Non-Responder (*n* = 107)	*p*-value
Gender, n (%)	Male	307 (65.3%)	51 (47.7%)	< 0.01
	Female	160 (34.0%)	55 (51.4%)	
	Missing	3 (0.6%)	1 (0.9%)	
Age, mean (SD)		54.5 (7.9)	57.8 (9.0)	< 0.01
Missing data for the age variable		9	12	
Working as a GP, n (%)	< 5 years	35 (7.4%)	–	
	5–10 years	95 (20.2%)	–	
	11–20 years	135 (28.5%)	–	
	> 20 years	203 (43.2%)	–	
	Missing	3 (0.6%)		
Number of GPs in practice, mean (SD)		2.10 (1.40)	–	
Number of physician’s assistants in practice, mean (SD)		3.94 (3.38)	–	
Number of home visits per week, mean (SD)		17.1 (16.2)		
Practice location, n (%)	Urban area	119 (25.3%)	–	
	Rural area	86 (18.3%)	–	
	Medium-sized town	253 (53.4%)	–	
	Missing	12 (2.6%)		
Positive attitude regarding task shifting, n (%) (missing data: 5)		283 (49.0%)	–	

*GP* General practitioner, *SD* Standard deviation

### Benefits and concerns

Table 2 shows different benefits and concerns regarding the implementation of the concept of task shifting. High benefits were described for having qualified staff (82.7%; CI = 79%, 86%), saving time with task shifting (95.2%; CI = 93%, 97%) and having more time for individual patients through task shifting (92.6%; CI = 90%, 95%). Moreover, 80% (CI = 77%, 84%) of the GPs indicated that task shifting could lead to more satisfaction. A small number of GPs (9.7%; CI = 7%, 12%) indicated that they were concerned about substituting in important aspects of health care, and 39% (CI = 34%, 43%) of the GPs were unclear about the responsibilities and legal aspects of task shifting.

**Table 2:** Benefits and concerns for the implementation of the concept of task shifting

Agreement regarding…	N (%)	95% CI
Time savings	436 (95.2%)	93% - 97%
More time for individual patients	425 (92.6%)	90% - 95%
Need for qualified staff	383 (82.7%)	79% - 86%
Increase in own satisfaction	362 (80.4%)	77% - 84%
Need for adequate payment for staff	284 (61.2%)	57% - 66%
Provision of health care for a larger number of patients	325 (73.7%)	70% - 78%
Lack of clarity about responsibilities and legal situations	180 (39.0%)	34% - 43%
Lack of financial incentive	176 (38.1%)	34% - 43%
Information loss due to the interface	149 (32.3%)	28% - 37%
Lack of clear definition of tasks for the different health care professionals	111 (24.0%)	20% - 28%
Lack of acceptance by patients	94 (20.3%)	17% - 24%
Substitutions in important aspects of health care	45 (9.7%)	7% - 12%
Competition against me as the GP	22 (4.8%)	3% - 7%

95% CI: Confidence interval

### Task shifting – Currently

Table 3 shows the results of currently delegated tasks. Currently, 281 (48.6%) of the GPs delegated at least seven tasks. The majority of these GPs (95.3%) delegated recording of an electrocardiogram, 92.6% delegated measuring blood glucose, 91.5% delegated taking blood, 88.9% delegated checking blood pressure, 86.2% delegated conducting spirometry tests and 70.4% delegated vaccinating. The tasks that were least delegated by these GPs were wound inspections (36.0%), advisory activities, such as diet and exercise (33.0%) and taking parts of the medical history (32.3%).

**Table 3:** Currently delegated tasks of GPs (n = 470)

Delegated tasks:	Yes	No
Record electrocardiogram	448 (95.3%)	17 (3.6%)
Measure blood glucose	435 (92.6%)	30 (6.4%)
Take blood samples	430 (91.5%)	35 (7.4%)
Take blood pressure	418 (88.9%)	47 (10.0%)
Conduct spirometry testing	405 (86.2%)	60 (12.8%)
Perform vaccination	331 (70.4%)	134 (28.5%)
Conduct standardized tests (e.g., Barthel index)	242 (51.5%)	223 (47.4%)
Inspect wounds	169 (36.0%)	295 (62.8%)
Provide advice (e.g., diet, exercise)	155 (33.0%)	309 (65.7%)
Take the patient’s medical history	152 (32.3%)	313 (66.6%)

### Prediction of factors for a positive attitude towards task shifting

The binary regression model regarding positive attitudes towards task shifting identified seven factors with a Nagelkerke R^2^ of 0.334 (Hosmer-Lemeshow Test *p* = 0.55), as presented in Table 4. Only significant results are shown. A positive attitude towards the concept of task shifting was significantly associated with increased job satisfaction (odds ratio (OR) = 4.36, confidence interval (CI) = 2.20, 8.64), the need for qualified staff (OR = 2.48, CI = 1.28, 4.82) and the probability of offering more health care to a larger number of patients (OR = 1.92, CI = 1.08, 3.41) and was negatively associated with unclear responsibilities and legal situations (OR = 0.47, CI = 0.28, 0.78) and information loss due to the interface (OR = 0.39, CI = 0.23, 0.67).

**Table 4:** Prediction of factors for positive attitudes towards task shifting – a binary-logistical regression model

Variables	OR (95% CI)	*p*-value
Increase in own satisfaction	4.36 (2.20–8.64)	< 0.01
Need for qualified staff	2.48 (1.28–4.82)	0.01
Provision of prospective advice regarding household remedies	2.12 (1.24–3.60)	0.01
Current inspection of wounds	2.11 (1.21–3.67)	0.01
Health care for a larger number of patients	1.92 (1.08–3.41)	0.01
Lack of clarity regarding responsibilities and legal situations	0.47 (0.28–0.78)	0.01
Information loss due to the interface	0.39 (0.23–0.67)	0.01

*OR* Odds ratio; 95% *CI* Confidence interval

## Discussion

Our results present an initial overview of experiences and attitudes towards the concept of task shifting from the perspective of GPs in a country beginning to change its attitudes towards task shifting. Our sample consisted of GPs with long work experience, nearly half of whom had positive attitudes towards shifting of tasks. In contrast to our non-responder sample, the participating GPs were younger and included a lower percentage of women. The concept of task shifting from the perspective of GPs was related to different important benefits but also to some concerns. The respondents strongly agreed that qualified staff and adequate payments for the staff are needed. As described in recent literature, the main barrier for high-performing team-based care, including task shifting, is the lack of an adequate remuneration of the work performed by non-clinical staff [9]. As our results demonstrated, GPs mainly delegated tasks that could be summarized under the term standardized procedures, such as recording electrocardiograms and measuring blood glucose, whereas fewer of the GPs delegated tasks that required communicative competence in terms of counselling, such as advisory activities. It could be assumed that GPs do not feel confident towards their staff concerning the performance of tasks that require communicative competence. It could also be assumed that there is a certain lack of awareness among the GPs regarding the tasks that can be delegated to their staff.

An important concern regarding the concept of task shifting, from the perspective of GPs, is that there is uncertainty regarding responsibilities and legal situations. A study on the extent of task shifting in 39 countries has shown that Germany is among a group of countries with no official legal regulations for task shifting from physicians to nurses in primary care [1]. Notwithstanding the above, as our results demonstrated, the proportion of different tasks that were currently delegated in our sample was very high. GPs strongly agreed that task shifting can lead directly to time savings and thus provide an opportunity to offer health care to more patients. These advantages of task shifting are comparable to findings in other studies [6, 8]. These findings have been complemented by an economic evaluation of the AGnES project and have shown that home visits performed by practice assistants result in time saving for GPs [5], thus allowing GPs to offer health care to more patients.

Therefore, it is necessary to invest in a well-performing primary care team performing different roles and comprising qualified staff. Changes in primary care are pending. The limited career development of practice staff has often been described [2, 9]. However, with the concept of task shifting, it is possible to appreciate the work of practice staff with new responsibilities that should be defined precisely. Multidisciplinary teams with different occupational backgrounds will be integral parts of primary care teams in order to meet the requirements of health care in the future and to reduce occupational stress and increase physicians’ job satisfaction. Different studies on job satisfaction have shown that GPs are often less satisfied with their working hours but are highly satisfied with their teams [16, 17]. As our results demonstrated, a positive attitude towards task shifting is associated with increases in job satisfaction and qualified staff members. Moreover, a recently published study has shown that task shifting to practice staff can lead to staff being more motivated and satisfied [18].

Our study provides an important contribution regarding the experiences and attitudes towards the concept of task shifting from the perspective of GPs in a country where the attitude towards task shifting is changing. The list of delegated tasks was developed from a literature review concerning the current state of the concept of task shifting in the German health system and within the international context. All participating GPs from the present study came from one federal state in Germany. Therefore, the findings are tentative, and it is not possible to determine cause-and-effect relationships. The strength of the study is that we included a non-responder analysis. Only GPs were involved in this study; non-physician staff, such as practice assistants, were not asked about their experiences with delegated tasks. The participation of GPs in this study was voluntary; therefore, a potential selection bias is likely. The questionnaire was sent to only GPs in a pilot trial but was not validated. Moreover, no power calculation was performed. Finally, this was a cross-sectional study; thus, we must be cautious about deriving causal links from these findings.

## Conclusions

German GPs are beginning to delegate tasks to non-physicians and should extend delegated tasks to their practice staff. As our study shows, GPs mainly delegated tasks that are standardized procedures. There is a willingness to expand the roles of non-clinical practice staff, but this willingness is dependent on legal requirements, adequate payment and the availability of qualified staff. There is a need for continuing professional development in primary care teams, especially for non-clinical practice staff.

## Additional file

Additional file 1: Questionnaire “Attitudes towards the concept of task shifting within general practice”. The questionnaire for the presented survey is available as additional file. (PDF 23 kb)

## Abbreviations

AGnES: General practitioner-supporting, community-based, e-health-assisted, systematic intervention
BBSR: Federal Institute for Research on Building, Urban Affairs and Spatial Development
CI: Confidence interval
GP: General practitioner
OR: Odds ratio
SD: Standard deviation
SPSS: Statistical package of social science
STROBE-Guidelines: Strengthening the Reporting of Observational Studies in Epidemiology
VERAH: Health care assistants in family practices
