# Supplementary material for: At the dawn of delegation? Experiences and attitudes of general practitioners in Germany – a questionnaire survey
Source: BMC Fam Pract. 2017 Dec 19;18:102. doi: 10.1186/s12875-017-0697-y (PMC5735503; doi:10.1186/s12875-017-0697-y)
Supplement: Additional file 1: — Questionnaire “Attitudes towards the concept of task shifting within general practice”. The questionnaire for the presented survey is available as additional file. (PDF 23 kb) [file 12875_2017_697_MOESM1_ESM.pdf]

UNIVERSITÄT ZU LÜBECK

# Attitudes towards the concept of task shifting within general practice

Dear colleagues,

Thank you for your participation. This survey is anonymous. Your answers will be analyzed from the Institute of Family Medicine, University Hospital Campus Luebeck. The information provided will be treated as strictly confidential. These data will not be released to third parties.

## Task shifting

**1. How is your attitude towards the concept task shifting?**

☐ Positive attitude ☐ Partly positive attitude ☐ Negative attitude

**2. Under which condition are you willing to shift tasks?** *(Multiple answers possible)*

☐ Not at all ☐ need for qualified staff

☐ Need for adequate payment for staff

☐ Other namely: ____________________

**3. Which tasks you already delegate?** *(Multiple answers possible)*

| | |
|---|---|
| Take the patient's history | ☐ |
| Measure of: | |
| - blood pressure | ☐ |
| - blood glucose | ☐ |
| Conduct/ record: | |
| - electrocardiogram | ☐ |
| - spirometry testing | ☐ |
| Take blood sample | ☐ |
| Perform vaccination | ☐ |
| Inspect wounds | ☐ |
| Conduct standardized tests (e.g. *Barthel-Index)* | ☐ |
| Provide advice (e.g. diet, exercise) | ☐ |
| Other namely: | |

**4. Task shifting can be lead to ...** *(Multiple answers possible)*

| | Yes | No |
|---|---|---|
| **a.** Time savings. | ☐ | ☐ |
| **b.** Provision of health care for a larger number of patients. | ☐ | ☐ |
| **c.** More time for individual patients. | ☐ | ☐ |
| **d.** Increase in own satisfaction. | ☐ | ☐ |

**5. Which concerns did you have towards task shifting?** *(Multiple answers possible)*

| | |
|---|---|
| **a.** Substitutions in important aspects of health care. | ☐ |
| **b.** Information loss due to the interface. | ☐ |
| **c.** Lack of acceptance by patients. | ☐ |
| **d.** Lack of clear definition of tasks for different health care professionals. | ☐ |
| **e.** Competition against me as the GP. | ☐ |
| **f.** Lack of clarity about responsibilities and legal situations. | ☐ |
| **g.** Lack of financial incentive. | ☐ |
| **h.** Other namely : | |

## Demograhic and practice characteristics

**6. How many GPs working in the practice?** |__|__| (number)

**7. How many physician‘s assistants working in the practice?** |__|__| (number)

**8. How many home visits will be done per week?** |__|__| (number)

**9. How old are you?** |__|__| years

**10. You are …** ☐ male ☐ female

**11. How many years did you working as a GP?** ☐ < 5 ☐ 5-10 ☐ 11-20 ☐ > 20 years

**12. In which district did you working?** *(Please indicate your location designation.)* |_|_|_|

**13. What kind of aspects did you missing in the survey?**

________________________________________

________________________________________

________________________________________

________________________________________

________________________________________

**Thank you for your participation!**
